# Trilostane: Beyond Cushing’s Syndrome

**DOI:** 10.3390/ani15030415

**Published:** 2025-02-02

**Authors:** Ali R. Olaimat, Parastoo Jafarzadehbalagafsheh, Mohammad Gol, Anna-Maria Costa, Giuseppe Biagini, Chiara Lucchi

**Affiliations:** 1 Laboratory of Experimental Epilepsy, Department of Biomedical, Metabolic and Neural Sciences, University of Modena and Reggio Emilia, 41125 Modena, Italy; ali.olaimat@unimore.it (A.R.O.); parastoo.jafarzadehbalagafsheh@unimore.it (P.J.); annamaria.costa@unimore.it (A.-M.C.); chiara.lucchi@unimore.it (C.L.); 2 Health Innovative Products and Technologies (HIP-TECH) Ph.D. Program, Department of Life Sciences, University of Modena and Reggio Emilia, 41125 Modena, Italy; 3 Radiology Department, South Tyneside and Sunderland NHS Foundation Trust, Sunderland SR4 7TP, UK; mohammad.gol@nhs.net; 4 Center for Neuroscience and Neurotechnology, University of Modena and Reggio Emilia, 41125 Modena, Italy

**Keywords:** allopregnanolone, brain, Cushing’s syndrome, epilepsy, neurosteroids, trilostane

## Abstract

Trilostane is a drug able to block the synthesis of progesterone from pregnenolone, dependent on the enzyme 3β-hydroxysteroid dehydrogenase/Δ^5−4^ isomerase. As a consequence of this effect, it is used to treat endocrinological diseases such as Cushing’s syndrome, especially in dogs. Because of the modulatory effects of trilostane on the hypothalamic–pituitary–adrenal axis, trilostane administration causes an increase in brain levels of neurosteroids with anticonvulsant properties, as in the case of allopregnanolone. Allopregnanolone is also of interest in curing depression, suggesting that trilostane might represent a tool to address neurological and psychiatric disorders. In this review, we investigated the historical development of this drug and its current use, mechanisms, and possible developments. By searching the literature from 1978 to 2025, we identified 101 papers describing studies with trilostane. Precisely, 55 were about dogs and trilostane, 3 were on cats, and 23 were with other animals. Some studies (15) were also designed with human patients. The main disease treatment with trilostane was hyperadrenocorticism. However, we also found two preclinical papers on trilostane’s potential use in psychiatric diseases and three on trilostane’s potential use in neurological disorders. Moreover, few clinical and preclinical studies suggested the involvement of neurosteroids modulated by trilostane in different neurological disorders, thus opening a possible new perspective for the use of this drug.

## Simple Summary

Drugs rarely are aimed at a single target. More frequently, drugs can affect the functioning of multiple systems as a consequence of the different levels of activity of the aimed target or because of the effects caused by drug interactions with the other possible targets. Trilostane is a veterinary drug used for very specific diseases, but the array of possible beneficial effects generated by trilostane could be useful for diseases different from those indicated for the use of this drug. Additionally, although abandoned as a drug to treat diseases in human patients, the use of trilostane in animals to address conditions still needing effective therapies could give trilostane appeal for a new appraisal in disorders common to humans and animals, such as epilepsy, anxiety, and depression. This review of the present evidence suggests that trilostane is a powerful tool to stimulate the production of hormones (steroids), making the brain more resilient to injuries and traumatic events, preventing more negative consequences caused by the triggered neuroinflammatory responses (a consequence common to seizures and, but not only, depression), which definitely could lead to more severe conditions.

## 1. Introduction

Trilostane, a synthetic steroid analogue first synthesized in the 1970s by Neumann and colleagues, was originally developed as an inhibitor of adrenal steroidogenesis due to its ability to competitively and reversibly inhibit 3β-hydroxysteroid dehydrogenase/Δ^5−4^ isomerase (3β-HSD). This enzyme is pivotal in the biosynthesis of corticosteroids, including cortisol and aldosterone [1].

Trilostane belongs to a class of steroidal heterocycles initially approved for the treatment of Cushing’s syndrome in humans in 1984. However, trilostane’s therapeutic use in humans was discontinued almost a decade later, and its U.S. Food and Drug Administration (FDA) approval was ultimately discontinued, due to inconsistencies in efficacy and concerns over safety profiles. Despite this, its approval for veterinary use by both the FDA and the European Medicines Agency (EMA) has marked trilostane as a cornerstone in the management of Cushing’s syndrome in dogs, where it remains widely utilized and well-studied for the treatment of pituitary-dependent and adrenal-dependent hyperadrenocorticism [2,3].

Beyond its established role in treating adrenal disorders, emerging evidence suggests that trilostane exerts a broader range of pharmacological effects, spanning diverse endocrine pathways and beyond. Recent studies have highlighted its influence on neurosteroidogenesis, inflammatory processes, and mood-related neurochemical dynamics, opening new avenues for its potential application in neurological, metabolic, and inflammatory disorders. Moreover, research into its effects on neurosteroids such as allopregnanolone and pregnenolone suggests significant implications for neuronal excitability, neuroprotection, and neurodegeneration. These findings present trilostane as a potential therapeutic agent for epilepsy, neurodegenerative diseases, and mood disorders, particularly in the veterinary context [4,5,6,7,8,9,10,11].

Given the overlapping pathophysiology of neurological and metabolic diseases in both human and animal models, trilostane’s expanded therapeutic applications warrant deeper investigation. Recent translational veterinary epilepsy research highlights the value of leveraging canine models to better understand drug-resistant epilepsy and explore innovative therapeutic strategies. These efforts not only provide insights into veterinary neurology but also improve our understanding of shared mechanisms and treatment approaches in human epilepsy [12]. In this review, we explore the pharmacological properties of trilostane, its mechanisms of action, and its clinical utility beyond the treatment of hyperadrenocorticism, with a particular focus on neurological disorders in veterinary medicine. Through a synthesis of current evidence, we aim to highlight trilostane’s multifaceted potential, emphasize existing knowledge gaps, and propose future research directions to unlock its full therapeutic capabilities.

## 2. Materials and Methods

A comprehensive literature search was conducted using the PubMed database to identify relevant articles on trilostane. The search term “trilostane” was used, and the search was limited to studies published between 1978 and 2025. Articles in English that addressed trilostane’s mechanism of action, pharmacology, clinical use, and veterinary applications were included; exclusion criteria involved single case reports, non-peer-reviewed materials, conference abstracts, and studies lacking full-text availability. The data from selected studies were extracted, synthesized, and organized into thematic sections to provide a thorough review of trilostane’s applications and properties.

## 3. Results

A total of 101 articles on trilostane were identified in the literature, including 55 studies on dogs, 3 on cats, and 23 on other animals, while 15 studies involved human patients. The primary therapeutic indication for trilostane was the management of hyperadrenocorticism. Additional studies explored its mechanism of action, pharmacokinetics, and its use in managing conditions such as alopecia X in dogs, equine Cushing’s syndrome, and Cushing’s syndrome in cats. Trilostane has also been studied for its applications in pain, inflammation, anxiety, depression, and hormonal regulation. In humans, it has been investigated for Cushing’s syndrome, hypertension, adrenal disorders, breast cancer, prostate cancer, and hepatocellular carcinoma. Importantly, some studies examined trilostane’s effects on neurosteroids, with potential applications in epilepsy. Furthermore, two preclinical studies suggested its potential use in anxiety, highlighting its diverse therapeutic applications.

### 3.1. Mechanism of Action

Trilostane is primarily used in the management of Cushing’s syndrome. Its therapeutic activity is attributed to its competitive and reversible inhibition of 3β-hydroxysteroid dehydrogenase/Δ^5−4^ isomerase (3β-HSD) in the adrenal cortex [13]. This enzyme is crucial for the biosynthesis of cortisol and aldosterone, as it catalyzes the conversion of 3β-hydroxysteroids to 3β-ketosteroids [14].

A study on healthy dogs suggested that trilostane affects the cortisone/cortisol ratio and modifies the expression of 11β-hydroxysteroid dehydrogenase (11β-HSD) enzymes in the adrenal glands. This may lead to the activation of 11β-HSD2 and/or inactivation of 11β-HSD1 [15].

In a study with male Wistar rats that examined the effect of trilostane on hepatic enzymes, it was found that trilostane decreased glucocorticoid receptor (GR) gene transcription and translation, increased 11β-HSD2 expression (the enzyme responsible for inactivating active glucocorticoids), had no effect on 11β-HSD1 (the enzyme responsible for activating glucocorticoids locally), and did not alter the expression of estrogen receptors (ER) or 3β-HSD enzymes. These findings suggest that trilostane primarily reduces the tissue response to glucocorticoids and mineralocorticoids rather than lowering their circulating levels [16].

Furthermore, a study on reproductive zebrafish revealed that trilostane affects targets beyond 3β-HSD, notably by altering gene transcription and regulatory networks [17].

### 3.2. Pharmacokinetics

Following oral administration, trilostane is efficiently absorbed in the gastrointestinal tract, with peak plasma concentrations observed within 1–2 h in dogs and 2–6 h in humans [18,19]. In rats, orally administered trilostane is metabolized into both conjugated and unconjugated forms, with conjugated metabolites excreted via bile and unconjugated forms via urine [20]. Human studies confirm rapid metabolism of trilostane to its primary active metabolite, 17-ketotrilostane. This conversion to ketotrilostane is both rapid and reversible, as evidenced by the presence of both compounds in plasma shortly after administration. Ex vivo studies have shown that 17-ketotrilostane is more effective in inhibiting cortisol and corticosterone secretion. In human pharmacokinetic studies, trilostane was eliminated from the bloodstream within 6–8 h, while nearly complete recovery of the drug and its metabolites occurred within 24–48 h through renal and biliary pathways [18,21]. In another pharmacokinetic study in rats, trilostane underwent reversible metabolism to form keto-trilostane. This study also calculated trilostane’s and keto-trilostane’s pharmacokinetic parameters following intravenous (IV) administration [22].

### 3.3. Management of Hyperadrenocorticism in Dogs

Trilostane has been extensively studied for its role in managing pituitary-dependent hyperadrenocorticism in dogs, demonstrating efficacy and safety. A clinical trial involving 78 dogs treated with trilostane for up to three years reported effective long-term management of the condition with no changes in adrenal size observed during the treatment period [23]. Similarly, a study of 30 dogs confirmed trilostane’s safety profile and efficacy in controlling pituitary-dependent hyperadrenocorticism [24]. Another study with 11 dogs observed changes in adrenal size and morphology after six weeks of treatment, with more significant alterations evident after at least one year of therapy [25].

Trilostane treatment has also been linked to notable endocrine and biochemical improvements. In one study involving 17 dogs with pituitary-dependent hyperadrenocorticism and 12 healthy controls, trilostane significantly reduced serum cortisol and aldosterone levels, though these levels remained higher than in the healthy group. Transient hyperkalemia was observed early in the treatment but resolved spontaneously [26]. Another study reported that trilostane reduced elevated parathyroid hormone and phosphate levels in dogs with pituitary-dependent hyperadrenocorticism after 30 weeks, though calcium levels increased significantly following treatment [27].

Several studies have evaluated trilostane in comparison with mitotane. While both are effective treatments for hyperadrenocorticism, trilostane is generally associated with fewer side effects. One observational study comparing the two treatments found no significant difference in survival times, though variations in data quality across centers may have influenced these findings [28]. Clemente et al. demonstrated that trilostane, when administered twice daily, significantly improved survival times in comparison to mitotane [29]. A study of 26 dogs confirmed similar efficacy between the two drugs in treating adrenal-dependent hyperadrenocorticism, but trilostane showed a higher safety profile [30]. Renie’s review of therapeutic profiles confirmed trilostane’s superiority in tolerability compared to mitotane [31].

Trilostane’s clinical benefits extend to metabolic and oxidative stress markers. In a study involving dogs with hyperadrenocorticism, trilostane reduced liver enzyme levels (alanine aminotransferase and alkaline phosphatase) and cholesterol but had minimal effect on bile acid concentrations after three months of treatment [32]. Additionally, trilostane has been shown to reduce oxidative stress markers, such as TBARS, to levels comparable to healthy dogs [33]. Despite its effectiveness in lowering cortisol levels, trilostane does not fully reverse immune suppression associated with hyperadrenocorticism, as observed in a study examining lymphocyte counts and subsets [34].

Haptoglobin levels, which are elevated in hyperadrenocorticism, were found to decrease significantly following trilostane treatment, indicating potential benefits for systemic inflammatory markers [35]. Lastly, trilostane treatment has been associated with improved survival rates in dogs diagnosed with pituitary-dependent hyperadrenocorticism compared to untreated dogs [36].

Overall, trilostane has emerged as a valuable therapeutic option for the management of hyperadrenocorticism in dogs. Its twice-daily regimen provides a tailored approach, effectively addressing clinical signs while minimizing side effects and improving survival outcomes.

#### Dosing and Monitoring in Dogs

The efficacy and safety of trilostane in managing pituitary-dependent hyperadrenocorticism have been widely studied. Research comparing single and twice-daily dosing of trilostane found both regimens to be equally safe, with bis-in-die dosing demonstrating greater clinical improvement [37]. A retrospective study reported slightly longer survival times for dogs treated with low-dose trilostane administered twice daily, compared to higher doses given once or twice daily [38]. Additionally, long-term administration of trilostane has demonstrated both safety and efficacy, with a study involving 44 dogs showing that twice-daily dosing enabled a lower total daily dose while maintaining clinical efficacy [39]. Research on nine dogs found that a single dose of trilostane reduced cortisol levels for a few hours but not at 24 h; switching to twice-daily dosing improved clinical outcomes and cortisol suppression [40]. Emerging evidence suggests that dosing regimens may influence trilostane’s effectiveness. A study involving dogs with pituitary-dependent hyperadrenocorticism noted that once-daily trilostane did not significantly reduce urinary catecholamines and metanephrines, likely due to insufficient cortisol suppression over 24 h [40]. Conversely, studies on naturally occurring hyperadrenocorticism support the benefits of twice-daily, low-dose trilostane regimens for optimal clinical outcomes and reduced side effects [41,42].

Individualized dosing has shown advantages over traditional body-weight-category-based dosing. A study found that tailoring trilostane doses per kilogram of body weight reduced side effects without compromising treatment efficacy [43]. Both trilostane and mitotane have been noted to reduce aldosterone secretory reserve in dogs with pituitary-dependent hyperadrenocorticism. Measurement of aldosterone levels 30 min post-adrenocorticotropic hormone (ACTH) stimulation identified more cases of reduced reserves than the traditional 60 min test, emphasizing the importance of timing in adrenal function assessment [44]. Timing of ACTH stimulation tests (ACTHst) is also critical, with studies recommending standardized timing after trilostane administration to ensure consistent results [45]. Another study highlighted the variability in cortisol measurements, showing significantly higher levels 9–12 h post-trilostane administration compared to 3–6 h, further stressing the need for standardized monitoring [46].

Alternative monitoring methods have also been explored. Measuring cortisol concentrations before and three hours after trilostane administration has been proposed as a reliable alternative to ACTHst for assessing the treatment response [47]. However, prepill cortisol measurements taken an hour apart demonstrated variability, influencing therapeutic decisions in 30% of cases. Stress during blood collection emerged as a potential factor, suggesting documentation of handling conditions to improve reliability [48].

Studies have shown that clinical signs and owner-reported symptoms do not reliably correlate with measurable hormone levels like cortisol or ACTH, emphasizing the need for objective laboratory tests over symptom-based monitoring [49]. This was supported by findings that ACTHst’s results often do not align with clinical signs of hyperadrenocorticism, underscoring the necessity for additional clinical tools for effective management [50]. Regular monitoring of ACTH, cortisol, and the cortisol/ACTH ratio has been identified as crucial for guiding dose adjustments and ensuring safe therapy, with basal plasma ACTH concentrations serving as a marker for preventing hypocortisolism during trilostane treatment [51].

Baseline cortisol measurements have been validated as a screening tool for routine monitoring in asymptomatic dogs undergoing trilostane therapy, providing a straightforward first-line approach [52]. However, studies confirm that ACTH stimulation remains essential for effective monitoring [53]. ACTH levels at diagnosis can also help determine starting trilostane doses, with higher levels correlating to higher dose requirements [54]. Despite extensive research, no single laboratory test has proven consistently reliable for monitoring trilostane therapy [55].

Incorporating haptoglobin, alanine aminotransferase, and gamma-glutamyl transferase, alongside clinical observations, may improve the monitoring of trilostane treatment efficacy, reducing reliance on the ACTH stimulation test [56]. However, baseline cortisol concentration alone has been found insufficient as a monitoring tool for pituitary-dependent hyperadrenocorticism treatment, particularly in twice-daily trilostane regimens, as it fails to predict excessive or insufficient cortisol suppression accurately [57].

Low-dose, twice-daily trilostane administration is effective and safer than high-dose, once-daily regimens [58]. This conclusion was corroborated by additional research emphasizing the improved safety profile of bis-in-die dosing [59]. Larger body weight has been associated with lower required doses of trilostane per kilogram to manage clinical signs, suggesting the need to reconsider manufacturer-recommended starting doses to align with clinical findings [60].

### 3.4. Other Veterinary Uses

#### 3.4.1. Alopecia X in Dogs

A study involving 24 dogs with alopecia X reported positive results in terms of hair regrowth. The authors suggested that these outcomes might be due to trilostane’s effect on reduced steroidogenesis or its interaction with hormonal receptors, with the latter being a subject of debate [61]. A compilation of three case reports on dogs with alopecia X suggests the potential effectiveness of trilostane in promoting hair regrowth [62].

#### 3.4.2. Equine Cushing’s Syndrome

A clinical study investigated the effect of trilostane in 20 horses with equine Cushing’s syndrome. Trilostane was found to be safe and well-tolerated. In terms of its efficacy, it was effective in managing the symptoms of the disease, although it did not reduce cortisol levels [63].

#### 3.4.3. Feline Cushing’s Syndrome

A compilation of five case reports on cats with pituitary-dependent hyperadrenocorticism highlights the potential of trilostane in regulating cortisol levels and alleviating symptoms. However, the findings should be interpreted cautiously due to the limited number of cases and the lack of a well-defined pharmacokinetic profile for the drug in cats [64].

A retrospective study of 15 cats with hyperadrenocorticism found that trilostane effectively improved clinical signs and was well-tolerated over long-term administration. In diabetic cats, better glycemic control was observed, with a significant reduction in insulin requirements [65].

Another retrospective study confirmed the positive effect of trilostane in managing pituitary-dependent hyperadrenocorticism in cats, particularly in improving their quality of life [66].

### 3.5. Trilostane in Humans

#### 3.5.1. Cushing’s Syndrome

A clinical study on patients with primary aldosteronism due to adrenal adenoma or bilateral adrenal hyperplasia confirmed the efficacy of trilostane in reducing serum aldosterone levels. Trilostane was generally well-tolerated, with only mild gastrointestinal side effects reported [67].

Another clinical study confirmed the safety of long-term trilostane use in idiopathic hyperaldosteronism in human patients, highlighting its reversible inhibitory effect on 3β-HSDH [68].

Engelhardt and Weber confirmed the lack of efficacy of trilostane in human Cushing’s syndrome patients, attributing it to the inconsistent response of cortisol to trilostane therapy [69,70]. As we mentioned earlier, trilostane authorization for human use has been revoked.

#### 3.5.2. Hypertension and Adrenal Disorders

Interestingly, a study utilized Dahl inbred salt-sensitive (SS/jr) rats and administered trilostane at a dose of 0.3 micrograms per hour through both intracerebroventricular and subcutaneous routes. While subcutaneous administration showed no impact on blood pressure, intracerebroventricular administration led to a reduction in blood pressure in rats on a high-salt diet. The authors attributed these findings to the influence of aldosterone synthesized in the brain. These results are consistent with previous research, which reported hypertensive effects only at significantly higher doses of 650 mg/kg [71]. In hypertensive dogs with pituitary-dependent hyperadrenocorticism, trilostane alone was insufficient to control systolic blood pressure, and many dogs required additional antihypertensive medications, such as benazepril and amlodipine, for effective management [72].

Also, a study in rats demonstrated the potential of trilostane to prevent potassium loss induced by the diuretic furosemide [73]. Another study demonstrated the potential of trilostane to delay potassium loss in rats and prevent it in dogs induced by the diuretic hydrochlorothiazide [74].

Interestingly, a comparison of six case reports for human patients suggests the potential use of trilostane for treating aldosterone-dependent hypertension [75]. Additionally, a clinical study in humans confirmed the ability of trilostane to reverse hypokalemia induced by hydrochlorothiazide by decreasing aldosterone synthesis [76].

#### 3.5.3. Other Trilostane Putative Molecular Targets Revealed by Studies on Hormone-Dependent Cancer

Trilostane has been studied for its potential activity as a treatment for advanced breast cancer in humans. It is believed that trilostane exerts its effect in advanced breast cancer through three mechanisms: (i) blocking the biosynthesis of estrogen, (ii) direct inhibition of estrogen receptor activity, and (iii) promoting the expression of β-estrogen receptors. These mechanisms collectively target hormone-driven tumor growth. Trilostane has undergone numerous clinical trials, as extensively reviewed by Puddefoot et al. [77]. However, Tueni et al. reported that trilostane does not interact with estrogen receptors or other major hormonal sex steroid receptors [78]. Additionally, Lino et al. confirmed that trilostane exerts its effect without interacting with estrogen receptors in mammary tumors induced in SD rats [79].

A study conducted on MCF-7 cell lines and intact uterus rats demonstrated that trilostane can up-regulate β-estrogen receptor expression and suppress specific estrogen-regulated genes, such as MMP-10, suggesting its potential in managing tamoxifen-resistant breast cancer [80].

An in vitro study on prostate cancer cell lines showed that trilostane inhibits the production of androstenedione, testosterone, and dihydrotestosterone (DHT) from dehydroepiandrosterone (DHEA) by targeting 3β-HSD. However, trilostane also acts as an androgen receptor agonist, indicating that it should be used with caution [81]. The same results were confirmed by another in vitro study [82].

Recently, Lin and colleagues reported the synergistic effect of combining trilostane with a low dose of sorafenib in the treatment of human hepatocellular carcinoma [83].

#### 3.5.4. Neuroinflammation: Pain and Epilepsy

Neurosteroids’ effects are well-documented across a range of pathological conditions, including epilepsy, stress, anxiety, depression, psychosis, ataxia, and pain [84,85]. A key mechanism through which neurosteroids exert their influence is by modulating the gamma-aminobutyric acid type A (GABA_A_) receptor, a major target involved in inhibitory signaling. Neurosteroids such as allopregnanolone, pregnanolone, and α-tetrahydrodeoxycorticosterone enhance GABA_A_ receptor function, thereby promoting inhibition [11]. Trilostane has demonstrated significant potential in reducing inflammation and modulating pain through its influence on neurosteroid production. Neurosteroids such as allopregnanolone, pregnanolone, and progesterone are critical regulators of neuronal excitability and inflammation, acting primarily through the modulation of GABA_A_ receptors. These receptors play a pivotal role in enhancing inhibitory signaling and reducing excitotoxicity, which are essential mechanisms for controlling both inflammation and pain [5,86].

Our previous study showed that repeated administration of trilostane significantly increased neurosteroid levels, particularly allopregnanolone, in the hippocampus and neocortex. This increase was associated with a marked reduction in microglial activation in the subiculum, an area of the brain often implicated in neuroinflammatory responses. Microglial activation is closely linked to the release of pro-inflammatory cytokines, which exacerbate inflammation and pain. By suppressing this activation, trilostane indirectly reduces inflammatory-driven pain. Trilostane has been shown to delay the onset of epileptogenesis in a kainic acid-induced epilepsy model, attributed to its remarkable ability to elevate allopregnanolone levels. This neurosteroid has well-established anti-inflammatory properties and contributes to the attenuation of chronic neuroinflammation, which often underlies persistent pain conditions [5].

In addition to its anti-inflammatory effects, trilostane exhibits analgesic properties in models of inflammatory pain. For instance, in the formalin test, which evaluates both early nociceptive and late inflammatory pain phases, trilostane significantly reduced pain-related behaviors during the late phase, underscoring its efficacy in modulating inflammatory pain. Its action may also involve the inhibition of key inflammatory mediators, such as tumor necrosis factor-alpha and monocyte chemoattractant protein-1, which are reduced in systemic and localized inflammation models [86].

#### 3.5.5. Trilostane in Anxiety and Depression

Trilostane has shown significant antidepressant- and anxiolytic-like effects in studies on mice, highlighting its potential role in treating mood disorders. Since trilostane modulates the biosynthesis of neuroactive steroids, such as pregnenolone, progesterone, DHEA, and their sulfate derivatives, which are key regulators of neurotransmitter systems, including glutamate, GABA, and serotonin (5-HT). These neurotransmitters play a critical role in mood regulation and are usually dysregulated in patients with anxiety and depression. In forced swimming tests, trilostane decreased immobility, an indicator of antidepressant activity, and synergized with selective serotonin reuptake inhibitors, such as fluoxetine and sertraline. Its effects were associated with increased hippocampal 5-HT, norepinephrine turnover, and modulation of the hypothalamic–pituitary–adrenal axis, as demonstrated by reduced stress-induced increases in corticosterone and ACTH levels. Furthermore, trilostane facilitated the antidepressant-like properties of neurosteroids like DHEA sulfate while paradoxically altering hippocampal and plasma steroid levels. In the elevated plus-maze test, trilostane increased open-arm exploration, reflecting reduced anxiety. These findings suggest that trilostane’s ability to regulate neurosteroid and monoamine dynamics, along with its influence on stress response systems, supports its potential as a possible therapeutic strategy for anxiety and depression [9,10].

### 3.6. Role in Hormonal Regulation

Clinical studies have highlighted trilostane’s influence on the hypothalamic–pituitary–testicular axis, revealing decreased testosterone levels coupled with increased luteinizing hormone. This disruption has implications for male sexual function [87]. In another study, healthy rats exhibited a significant reduction in testosterone levels, while humans showed impaired testosterone synthesis during trilostane treatment [88]. During pregnancy in rabbits, trilostane administration led to lower progesterone levels, resulting in premature delivery [89].

Moreover, trilostane pretreatment has proven to enhance the efficacy of misoprostol in mid-trimester pregnancy terminations in humans by lowering progesterone levels with tolerable side effects [90,91]. Additionally, in vitro studies indicate selective inhibition of 3β-HSD in adrenal glands but limited effects in the corpus luteum. Despite its inhibitory action on ovarian progesterone synthesis in various species, its impact on dogs remains limited, as it fails to terminate mid-term pregnancies even after seven days of treatment [92,93,94].

In vitro research using sheep and human adrenal tissues (from patients with Cushing’s or Conn’s syndrome) demonstrated that trilostane enhances 11β-hydroxysteroid dehydrogenase activity in sheep, decreasing cortisol while increasing cortisone levels. However, this effect was absent in human tissues [95].

Hormonal disruptions were observed in dogs with pituitary-dependent hyperadrenocorticism, where pre- and post-ACTH stimulation results showed significant changes in cortisol, aldosterone, and other adrenal-related hormones over time (Table 1) [96].

Also, the 24 h effects of the first dose of trilostane on hormones were investigated and summarized in Table 2 [97].

In aquatic species, trilostane was shown to disrupt steroid production in the male Japanese rice fish, medaka, triggering compensatory genetic responses that interfered with normal hormonal regulation.

Finally, comparisons between healthy dogs and those with pituitary-dependent hyperadrenocorticism revealed significant differences in cortisol and cortisone levels. Baseline cortisol remained stable during trilostane therapy, but cortisone levels decreased over time, resulting in a lower cortisol/cortisone ratio post-ACTH stimulation [98].

### 3.7. Safety and Side Effects

Trilostane is generally well-tolerated in healthy individuals, with clinical studies showing a minimal impact on the normal function of the hypothalamic–pituitary–adrenal axis [99]. Research on healthy male rats has revealed that trilostane increases adrenal weight and induces morphological changes in the adrenal glands, particularly affecting adrenocortical cells [100,101]. Similarly, studies in both healthy and hypertensive rats reported changes in adrenal weight and morphology, with higher trilostane doses being associated with elevated blood pressure in both groups [102]. These findings of adrenal enlargement were further corroborated in additional studies involving rats [103].

In dog studies, trilostane therapy has demonstrated comparable effects. In dogs with pituitary-dependent hyperadrenocorticism, it caused adrenal gland enlargement and notable morphological changes [104]. Furthermore, in a study of 21 healthy dogs treated with trilostane, findings suggested a potential stimulation of pituitary tumor growth, potentially accelerating corticotroph adenomas in dogs already diagnosed with Cushing’s disease [105]. Interestingly, a study in rats demonstrated that adrenal necrosis during trilostane therapy is caused by elevated ACTH levels rather than by trilostane itself, highlighting the complexity of its endocrine effects [106].

Although iatrogenic hypoadrenocorticism is an uncommon side effect of trilostane therapy, it is often transient and resolves spontaneously in most cases. However, it can be life-threatening, particularly when triggered by concurrent illnesses in dogs undergoing treatment [107]. Post-treatment evaluations in dogs with pituitary-dependent hyperadrenocorticism have shown that while iatrogenic hypocortisolemia is unpredictable, it typically resolves within six months [108]. Additionally, hypoadrenocorticism associated with trilostane is not strongly dose-dependent, with approximately 75% of cases resolving spontaneously [109].

### 3.8. Neurosteroids, Trilostane, and the Current Evidence: A Possible Role in Neurodegenerative Diseases?

Neurosteroids, a class of steroids synthesized from cholesterol in the central and peripheral nervous systems, were first described in 1981 [110]. These compounds influence neuronal cells by modulating their function and activity [111]. Their primary mechanism of action involves allosteric modulation of GABA_A_ receptors [11,112]. Neurosteroids play a crucial role in regulating neuroinflammation, which is a common characteristic of neurodegenerative diseases [113]. In particular, these compounds can inhibit microglial activation and decrease the production of proinflammatory cytokines, offering a mechanism by which they modulate neuroinflammation [113]. Microglia, along with macrophages and lymphocytes, contain GABA_A_ receptors, which, when activated by allopregnanolone, can reduce the release of pro-inflammatory mediators [114,115]. This process helps suppress nuclear factor kappa–light–chain–enhancer of activated B cells (NF-κB) activation and inflammatory molecule production [116,117].

Neurosteroids are implicated in various neurological conditions, including epilepsy, anxiety, and neurodegenerative and other brain disorders [118]. Recently, our laboratory observed reduced levels of neurosteroids in the cerebrospinal fluid of amyotrophic lateral sclerosis human patients [8]. Additionally, altered levels of neurosteroids, specifically allopregnanolone and pregnanolone, were identified in Parkinson’s disease patients with GBA mutations, suggesting a correlation between these changes and disease severity [4].

**Figure 1:**
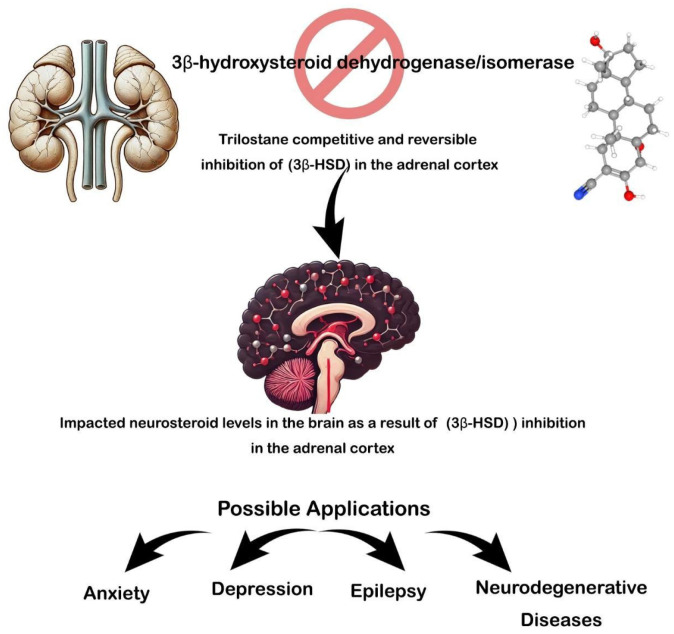
Possible applications of trilostane in neuronal diseases.

Interestingly, our lab reported that administering two injections of trilostane significantly increased the levels of pregnenolone, progesterone, 5α-dihydroprogesterone, and allopregnanolone in the hippocampus and neocortex of healthy rats (Figure 1) [7]. Moreover, trilostane has a positive effect by antagonizing epileptogenesis in rats [5,6,7].

As regards neurodegenerative diseases, different studies have revealed significant similarities between cognitive dysfunction in dogs and human Alzheimer’s disease, as well as between Alzheimer’s disease in humans and most other primates [119,120,121]. This similarity begins with changes in brain morphology, followed by the appearance of disease signs and symptoms [122,123,124,125]. Also, in dogs, the most prominent signs observed are memory loss, disorientation, behavioral changes, and confusion [126].

Interestingly, impaired mitochondrial structure and function have been reported in AD [127]. Also, in amyotrophic lateral sclerosis, recent data demonstrated alterations in mitochondrial shape, energy production, and calcium regulation [128]. It is well known that mitochondria are vital for neurosteroids’ early biosynthesis step [129]. Since these molecules are potent regulators of brain functions and impact various biological processes in neuronal cells, a link between this organelle malfunction and depleted neurosteroid levels may be proposed. Interestingly, one of the most prominent neurosteroids, allopregnanolone, is currently being studied as a potential treatment for fragile X-associated tremor/ataxia syndrome [130]. The enzyme 17β-hydroxysteroid dehydrogenase type 10 (17β-HSD10), which inactivates allopregnanolone [131], is found to be elevated in the brains of individuals with Alzheimer’s disease and corresponding animal models [132]. Regarding amyotrophic lateral sclerosis, the exogenous administration of progesterone and allopregnanolone in the Wobbler mouse model counteracts motor neuron degeneration, suggesting that these neurosteroids play an important role in its pathophysiology [133,134]. Compensation for depleted neurosteroids with trilostane treatment may enhance neuronal survival and offer a potential therapeutic strategy to slow down or even reverse the progression of neurodegenerative diseases in dogs.

## 4. Discussion

As we explore the potential of trilostane in treating veterinary neurological disorders, it becomes clear that further research is essential to unlocking its full therapeutic potential. While trilostane has been studied extensively in the context of adrenal-related conditions, its role in neurological disorders in animals, particularly in epilepsy and neurodegenerative diseases, remains underexplored. The promising effects of trilostane on modulating steroidogenesis and influencing neurosteroid levels suggest it could have significant implications for managing conditions like canine epilepsy, which affects a notable portion of the pet population (ranging from 0.5% to 5.7% prevalence) [135]. Given the complex interactions of neurosteroids with the nervous system and their influence on neuronal excitability, more focused studies are needed to understand the precise mechanisms through which trilostane can modulate brain activity and potentially offer a new avenue for treatment (Figure 1).

## 5. Conclusions

We urge researchers to consider the acute administration of trilostane as a potential tool for treating neurological conditions in dogs and other animals, particularly given its effects on epilepsy and epileptogenesis, which impact a large number of pets. Our previous studies have documented evidence of trilostane’s effects on epilepsy and epileptogenesis in a rat model, primarily by modulating neurosteroid levels in the brain. With further investigation into its safety profile, mechanisms of action, and efficacy in treating neurological disorders, trilostane could become an important addition to veterinary therapeutic strategies. A multidisciplinary approach that combines clinical trials and laboratory studies will be essential for gaining a9 deeper understanding of trilostane’s role in veterinary neurology and its potential benefits for animals suffering from neurological diseases.

## Figures and Tables

**Table 1:** Effect of trilostane on hormone release after 1–2 or 3–7 weeks of treatment in dogs with pituitary-dependent hyperadrenocorticism before and after adrenocorticotropic hormone (ACTH) stimulation test [96].

Hormone	Effect of Trilostane Treatment	Post-ACTH Stimulation
Cortisol	Decreased significantly	Decreased significantly
Aldosterone	Increased significantly	Decreased significantly
17-OH-pregnenolone	Increased significantly	Increased significantly
Dehydroepiandrostenedione	Increased significantly	Increased significantly
17-OH-progesterone	No change	No change
Androstenedione	No change	No change
21-deoxycortisol	No change	Decreased significantly
11-deoxycortisol	Increased significantly	No change
Endogenous ACTH	Increased significantly	-

**Table 2:** Effects of the first dose of trilostane on hormone levels in dogs with pituitary-dependent hyperadrenocorticism within 24 h [97].

Endocrine Alterations	Effect of Trilostane First Dose	Latency to the Effect (h)
Cortisol	Decreased significantly	2–4
Endogenous ACTH	Increased significantly	3–12
Aldosterone	Increased significantly	16–20
Renin activity	Increased significantly	6–20
Potassium (serum)	Decreased significantly	0.5–2
Sodium (plasma)	No changes	-
Free calcium (plasma)	No changes	-
